# Serum Interferon-Related MicroRNAs as Biomarkers to Predict the Response to Interferon Therapy in Chronic Hepatitis C Genotype 4

**DOI:** 10.1371/journal.pone.0120794

**Published:** 2015-03-19

**Authors:** Tarek Kamal Motawi, Olfat Gamil Shaker, Shohda Assem El-Maraghy, Mahmoud Ahmed Senousy

**Affiliations:** 1 Department of Biochemistry, Faculty of Pharmacy, Cairo University, Cairo, Egypt; 2 Department of Medical Biochemistry and Molecular Biology, Faculty of Medicine, Cairo University, Cairo, Egypt; National Taiwan University Hospital, TAIWAN

## Abstract

MicroRNAs are messengers during interferon-virus interplay and are involved in antiviral immunity, however, little is known about interferon-related microRNAs regarding their detection in serum and their potential use as non-invasive diagnostic and prognostic biomarkers in chronic hepatitis C (CHC). To elucidate some of the molecular aspects underlying failure of pegylated interferon-α/ribavirin therapy, we investigated pretreatment expression profiles of seven selected interferon-related microRNAs (miR-146a, miR-34a, miR-130a, miR-19a, miR-192, miR-195, and miR-296) by quantitative RT-PCR custom array technology in serum of Egyptian CHC genotype 4 patients and whether their pretreatment levels would predict patient response to the combination therapy. One hundred and six CHC patients and forty matched healthy controls were included. Patients were divided into sustained virological response (SVR) and non-responder (NR) groups. Serum miR-34a, miR-130a, miR-19a, miR-192, miR-195, and miR-296 were upregulated, whereas serum miR-146a was downregulated in CHC compared to controls. Significant correlations were found between expression levels of studied microRNAs and also with clinical data. Pretreatment levels of miR-34a, miR-130a, and miR-195 were significantly higher, whereas miR-192 and miR-296 levels were significantly lower in SVR than NR patients. miR-19a and miR-146a levels were not significantly different between the two groups. miR-34a was superior to differentiate CHC from controls, whereas miR-296 was superior to discriminate SVR from NR patients by receiver operating characteristic analysis. Multivariate logistic analysis revealed miR-34a and miR-195 as independent predictors for SVR and miR-192 as an independent variable for non-response. In conclusion, pretreatment expression profiles of five interferon-related microRNAs are associated with treatment outcome in CHC. Of these, miR-34a, miR-195, and miR-192 could predict treatment response. The profiling results could be used as novel non-invasive diagnostic and prognostic pharmacogenetic biomarkers for treatment personalization in CHC and could help to identify new microRNA-based antivirals.

## Introduction

Eradication of hepatitis C virus (HCV) is a global public health problem. HCV is a leading cause of chronic hepatitis, cirrhosis, and hepatocellular carcinoma as well as the most common indication for liver transplantation worldwide [1]. Egypt is plagued by the largest overwhelming HCV epidemic in the world with the highest HCV prevalence (14.7%) nationwide [2]. The HCV incidence in Egypt is very high, ranging from 0.8 to 6.8/1,000 persons annually [2], with an estimated 168 600 new infections occurred in 2013 [3]. HCV-4 is the predominant genotype in Egypt and responsible for at least 91% of infections leading to progressive HCV-related liver disease [4].

Until the emergence and approval of an effective interferon (INF)-free regimen, pegylated interferon (PEG-INF)-α will remain an integral part of the treatment of HCV-4 [5]. Newer treatment regimens, including direct acting antivirals in combination with PEG-INF-α and ribavirin (RBV) are more effective against HCV genotype 1, with higher rates of sustained virological response (SVR) in treatment-naïve patients reaching 75% [6]. However, these regimens are associated with poor response in prior null responders especially those with cirrhosis and are challenged by new adverse events and non-eligibility for many patients [7,8]. At present, these new regimens remain largely untested in HCV-4 populations, and the current national standard of care therapy comprises PEG-IFN-α along with RBV [9], with associated SVR rates of more than 60% [10]. INF-based regimens are compromised with high pill burden and undesirable adverse effects [10]; therefore, there is a desperate need to predict failure of response.

MicroRNAs (miRNAs) are small ~22 nucleotide non-coding RNAs that deregulate gene expression by mRNA degradation, or translational repression. Cellular miRNAs can regulate diverse domains, including proliferation, differentiation, immune reaction, and tumorigenesis. Circulating miRNAs display consistent profiles between healthy individuals and significantly altered expression in diseases; however, the functions of these miRNAs remain to be elucidated [11]. They may be released due to tissue damage such as miR-122 and miR-192 in liver injury [12], or mediators of cell-cell communication [11]. The high stability of miRNAs in serum and their sensitive detection by quantitative PCR established their potential role as non-invasive diagnostic biomarkers for liver injury, or predictive of liver disease progression, or treatment outcome in HCV infection [13].

Endogenous IFNs are antiviral and immunomodulatory cytokines that trigger the janus kinase/signal transducer and activator of transcription (JAK/STAT) signalling with subsequent induction of INF-stimulated genes (ISGs), including both protein-coding and miRNA genes [14]. Evidence suggests that RNA interference through miRNAs is an inherent component of the IFN-antiviral arsenal [14,15]. Specific IFN-β-induced miRNAs (miR-196, miR-296, miR-351, miR-431, and miR-448), in conjunction with INF-β-mediated downregulation of miR-122, may attenuate HCV replication in a sequence specific manner [15]. miRNAs also regulate INFs, INF-signaling, and ISGs suggesting a complex interplay between INF and miRNAs [14]. On the other hand, HCV can alter the expression profiles of miRNAs to sustain its replication [16,17]. HCV modulated INF-regulated miRNAs in HCV replicon clones, suggesting that HCV can control the INF defense-miRNA machinery [18]. The relationship between HCV infection and INFs and miRNA expression could shine a better understanding of the genetic factors involved in the failure of IFN-based therapy.

Molecular profiling is uprising as a pharmacogenetic tool to predict treatment outcome in chronic hepatitis C (CHC) as a way towards personalized therapy [19]. Emerging evidence suggests that miRNAs have an intense impact on the clinical outcome of standard INF therapy. Pretreatment hepatic miRNA expression pattern in CHC patients revealed that the expression levels of miR-34b, miR-145, miR-143, miR-652, and miR-18a were significantly higher in non-responder (NR) than SVR patients, while miR-27b, miR-422b, and miR-378 expression levels were higher in SVR than NR patients [20]. Increased pretreatment levels of serum miR-122 were correlated with early response and SVR [21], while reduced hepatic baseline miR-122 levels were associated with poor response to INF therapy [22]. INF-induced miRNAs (miR-1, miR-30, miR-128, miR-196, and miR-296) were reported to be differentially expressed in peripheral blood mononuclear cells (PBMCs) from CHC genotype 1 or 2 patients and might affect the response to INF therapy [23]. However, little is known about INF-related miRNAs regarding their detection in serum, their correlation with clinical data, and their potential use as non-invasive diagnostic and prognostic biomarkers in CHC.

Thus, in order to gain further insights into the genetic factors that may affect treatment outcome in CHC; it is interesting to examine pretreatment expression profiles of circulating INF-related miRNAs, in particular, miR-146a, miR-34a, miR-130a, miR-19a, miR-192, miR-195, and miR-296, previously reported to be induced by INFs in different *in vitro* models [15,17,18,24–26]. We investigated the expression profiles of these selected miRNAs as novel biomarkers in serum of CHC genotype 4 patients, their correlations with clinical data, and whether their pretreatment levels would predict patient response to PEG-IFN-α/RBV therapy. Our study revealed that the profiling results could be implicated as novel non-invasive diagnostic and prognostic pharmacogenetic biomarkers for treatment personalization in CHC and could also be used to identify new miRNA-based antiviral therapeutics.

## Materials and Methods

### Patients

One hundred and six Egyptian patients with CHC and 40 healthy controls were included in this study. CHC patients were admitted at the outpatient’s clinic of the liver unit, Tropical Medicine Department, at Kasr El-Aini Hospital, Cairo University, Cairo, Egypt, to receive standard PEG-IFN-α/RBV therapy. The diagnosis of CHC was made by serum biochemical and serological tests and peripheral blood cell counts as well as histological findings in liver biopsy specimens showing evidence of chronic hepatitis. All the patients were anti-HCV positive with detectable serum HCV RNA genotype 4. All patients were treatment-naïve and never previously received IFN therapy, immunomodulatory, or hepatoprotective treatment.

Patients presented with liver cirrhosis, or hepatocellular carcinoma, autoimmune hepatitis (presence of antinuclear antibody titer >1/160), alcohol-induced liver injury, hepatitis B virus (HBV) antigen or antibody, human immunodeficiency virus infection, active schistosomiasis, hypertension, renal insufficiency, proteinuria, suspected infections, clinically overt diabetes mellitus, thyroid dysfunction, or any other endocrine disorder or hematological disorder; hemoglobin (<11 g/dl), total leukocyte count (<3,000/mm^3^), neutrophils (<1,500/mm^3^), platelets (<100,000/mm^3^) or any disease other than CHC infection were excluded.

A total of 40 healthy control volunteers were sex and age matched to the patient population. All had completely normal liver function tests, normal ultrasound of the liver and biliary system, and negative serological findings for viral and autoimmune liver diseases and diabetes. Written informed consent was obtained from all patients and controls for gene analysis. The study protocol and informed consent were approved by the ethics committee of the Faculty of Pharmacy, Cairo University and conformed to the ethical guidelines of the Helsinki Declaration.

### Treatment regimen

Each patient received PEG-IFN-α2b, at 1.5 μg/kg body weight once a week by subcutaneous injection combined with daily oral weight-based ribavirin at 600, 800, or 1000 mg/day for patients with a body weight of less than 60 kg, between 60 and 80 kg, and more than 80 kg, respectively, for 48 weeks. Patients were prospectively followed and all of them were seen, at least, at weeks 4, 12, 24, and 48 while on treatment and 24 weeks after completing therapy.

### Laboratory assays and histological investigations

Venous blood samples were collected from each patient for routine pretreatment workup including, complete blood picture, serum alanine aminotransferase (ALT), aspartate aminotransferase (AST), alkaline phosphatase (ALP), albumin, total bilirubin, direct bilirubin, alpha-fetoprotein (AFP), and thyroid stimulating hormone using commercially available assays. Anti-HCV titers, anti-schistosomal antibodies, hepatitis B surface antigen, and hepatitis B core antibodies were assessed by routine methods using commercially available assays. Antinuclear antibody and anti-DNA were done using commercially available immunofluorescence kits. HCV patients were subjected to abdominal ultrasound and liver biopsy was taken from each patient before the onset of therapy to estimate the grade of activity and fibrosis according to Metavir Scoring System [27].

### HCV RNA and genotyping

Serum HCV RNA was extracted using viral RNA extraction kit (Qiagen, Valencia, CA, USA) and quantified prior to therapy and serially during therapy using a real-time RT-PCR kit (TaqMan assay reagents and Ambion, the RNA Company-one step RT-PCR kit, USA). HCV RNA genotyping was performed before treatment on the basis of the sequence of the core region using Ohno method. This method depends on nested PCR amplification of HCV core gene using genotype specific primers [28].

### Patients’ classification

All patients continued treatment for a total duration of 48 weeks and classified based on the final virological response to PEG-INF-α/RBV therapy. We excluded patients who relapsed and those who voluntarily dropped out or discontinued therapy due to severe adverse events. Thus, the study included a total of 69 patients who achieved SVR, which was defined as negative HCV RNA by PCR at the end of combination therapy and remains undetectable six months following completion of treatment in addition to 37 virological non-responder (NR) patients who failed to attain a negative HCV RNA by PCR at week 48.

### Serum miRNAs assay

#### RNA extraction

Total RNA containing miRNAs was extracted by miRNeasy extraction kit (Qiagen, Valencia, CA, USA) from 100 μl pretreatment serum samples using 500 μl QIAzol lysis reagent and incubated for 5 min at room temperature. 100 μl chloroform was added, mixed by vortex for 15 s, incubated for 2–3 min at room temperature and then centrifuged at 12,000 xg at 4°C for 15 min. The upper aqueous phase was transferred to a new collection tube and 1.5 times its volume of 100% ethanol was added. 700 μl of this mixture was placed in RNeasy Mini spin column in 2 ml collection tube and centrifuged at 8,000 xg at room temperature for 15 s. After the mixture had completely passed the column, 700 μl of buffer RWT was added to each column, and again centrifuged at 8,000 xg at room temperature for 15 s. Then, 500 μl buffer RPE was added to the column and centrifuged at 8,000 xg at room temperature for 15 s. Another 500 μl buffer RPE was added to the column and centrifuged at 8,000 xg at room temperature for 2 min. The column was transferred to a new 1.5 ml collection tube and 50 μl RNase-free water was pipetted directly onto the column and centrifuged for 1 min at 8,000 xg to elute RNA. The quality of RNA was determined using nanodrop (Thermo scientific, USA).

#### Reverse Transcription (RT)

RT was carried out on 100 ng of total RNA in a final volume of 20 μl RT reactions (incubated for 60 min at 37°C and 5 min at 95°C) using the miRNeasy Reverse Transcription kit (Qiagen, Valencia, CA, USA) according to the manufacturer’s instructions.

#### Quantitative real-time PCR

Expression of mature miRNAs, including hsa-miR-146a-5p, hsa-miR-34a-5p, hsa-miR-130a-3p, hsa-miR-19a-3p, hsa-miR-192–5p, hsa-miR-195–5p, and hsa-miR-296–5p was evaluated by quantitative RT-PCR analysis using miScript miRNA PCR custom array (Qiagen, Valencia, CA, USA) according to the manufacturer’s protocol. The housekeeping miScript PCR control, miRNA SNORD68 was selected as internal control. SNORD68 was reported to be expressed stably in different tissues [29] and of comparable stability to other small non-coding RNAs in several tumors [30] making its possible use as normalization control in miRNA PCR analysis. Indeed, there are no reported results that SNORD68 alters its expression in HCV-infected patients or by IFN treatment. For real-time PCR analysis of each miRNA, 2.5 μl of diluted RT products (cDNA template) was mixed with 5.5 μl RNase free water, 10 μl QuantiTect SYBR Green PCR Master Mix and 2 μl miScript universal primer (reverse primer) (Qiagen, Valencia, CA, USA), and then added to a custom Rotor-Disc 100 Format R miRNA PCR array which contains miRNA-specific miScript primer assays. The Rotor-Disc was sealed with optical thin wall strips. Real-time PCR was performed using a Rotor gene Q Real Time PCR System (Qiagen, Valencia, CA, USA) with the following conditions: 95°C for 30 min, followed by 40 cycles at 94°C for 15 s, 55°C for 30 s, and 70°C for 30 s. The cycle threshold (Ct) is defined as the number of cycles required for the fluorescent signal to cross the threshold in real-time PCR. ΔCt was calculated by subtracting the Ct values of miRNA SNORD68 from the Ct values of the target miRNAs. MicroRNA relative expression levels were calculated by 2^-ΔCt^ and reported as normalized individual data points [31].

### Statistical analysis

Values were expressed as mean ± standard deviation (SD), median (25%-75% percentiles), or number (percentage) when appropriate. According to data normality, comparison of independent samples from two groups was performed using Student’s t test or the Mann—Whitney *U*-test when appropriate. Because data were not normally distributed, comparisons of serum miRNA expression levels and viral load were performed by applying Mann—Whitney *U*-test. To compare categorical data, a Chi squared (*X* ^*2*^) test, or Fischer exact test was performed. Receiver operating characteristic (ROC) analysis was performed to assess the diagnostic and prognostic accuracy and the area under the curve (AUC) was calculated. Logistic regression analysis was performed to identify predictors of treatment outcome. Data that were significant according to univariate analysis were then entered into multivariate analysis to determine the independent variables that affected the response. Associations between parameters were determined by Spearman correlation. We considered *P* to be significant at <0.05 with a 95% confidence interval (CI). All statistical analyses were performed using computer program Statistical Package for the Social Science (SPSS, Chicago, IL, USA) software version 15 for Microsoft Windows and GraphPad Prism 5.0 (GraphPad Software, CA, USA).

## Results

### Demographic and clinical features of CHC patients and healthy controls and treatment outcome of PEG-IFN-α2b/RBV therapy

Demographic and clinical data of the patients and controls are given in Table 1. Serum HCV RNA genotyping revealed that all patients were genotype 4a. CHC patients showed a significant increase in ALT, AST, ALP, total and direct bilirubin (each, *P*<0.0001), and AFP (*P*<0.001) amounting to 198.8%, 188.6%, 252%, 138%, 333%, and 176% of normal control values, respectively. On the other hand, serum albumin level showed a significant decrease reaching to 70.2% of normal control values (*P*<0.0001) (Table 1).

**Table 1:** Demographic and clinical data of CHC patients and healthy controls.

Parameter	Controls (n = 40)	CHC patients (n = 106)	*P* value
Sex			0.099
Male, n (%)	24 (60%)	80 (75.5%)	
Female, n (%)	16 (40%)	26 (24.5%)	
Age (years)	41.24±7.4	44.14±8.75	0.074
Range (years)	(22–57)	(22–60)	
ALT (IU/l)	29.67±7.37	59±25.5	<0.0001*
AST (IU/I)	29.48±6.39	55.6 ±22.8	<0.0001*
ALP (IU/l)	43.1±8.76	108.75±52.2	<0.0001*
Total bilirubin (mg/dl)	0.52±0.12	0.72±0.24	<0.0001*
Direct bilirubin (mg/dl)	0.14±0.06	0.46±0.35	<0.0001*
Albumin (g/dl)	5.66±1.58	3.97±0.72	<0.0001*
AFP (ng/ml)	2.78±0.94	4.9±3.66	0.0007*

Data are expressed by mean ± SD, or number (percentage) for sex.

* indicates statistical significance (*P*<0.05).

According to final response to combination therapy, patients were divided into 69 who attained SVR (65%) and 37 NR patients (35%). Patients with SVR showed viral clearance revealed by undetectable HCV RNA at the end of treatment and 6 months after completion of combination therapy. SVR and NR patients showed no significant difference regarding sex, age as well as pretreatment levels of albumin, total and direct bilirubin, hemoglobin, total leukocyte count, and fibrosis stage. Patients with SVR exhibited significantly lower pretreatment levels of ALT (*P*<0.05), AST, ALP, AFP (each, *P*<0.01), HCV RNA (*P* = 0.001), Metavir activity score (*P* = 0.001) and significantly higher platelet count (*P*<0.01) than NR patients (Table 2).

**Table 2:** Clinical, virological, and histological data of SVR and NR patients to PEG-INF-α2b/RBV therapy.

Parameter	SVR (n = 69)	NR (n = 37)	*P* value
Sex			1
Male, n (%)	52 (75.36%)	28 (75.67%)	
Female, n (%)	17 (24.36%)	9 (24.32%)	
Age (years)	43.90 ±9.37	44.93±6.85	0.7
Range (years)	(22–60)	(30–56)	
ALT (IU/l)	54.87±24.89	66.78±25.13	0.02*
AST (IU/I)	51.37±18.26	63.5±26.8	0.007*
ALP (IU/l)	98.7±45.4	127.5±56.6	0.005*
Total bilirubin (mg/dl)	0.75±0.29	0.74±0.27	0.619
Direct bilirubin (mg/dl)	0.43±0.22	0.53±0.53	0.171
Albumin (g/dl)	4.05±0.65	3.85±0.83	0.174
AFP (ng/ml)	4.12±3.16	6.33±4.12	0.003*
Hemoglobin (g/dl)	16.27±7.26	13.9±1.2	0.42
Total leukocyte count (x10^3^/mm^3^)	6.53± 2.15	6.3 ± 2.05	0.596
Platelet count (x10^3^/mm^3^)	219.8±62	189.6±41.3	0.009*
Viral load (x 10^5^ IU/ml)	2 (0.09–7.98)^a^	7.51 (1.1–30.75) ^a^	0.001*
Fibrosis stage, n (%)			0.13
F1	39 (56.5%)	14 (38%)	
F2	20 (29%)	13 (35%)	
F3	10 (14.5%)	10 (27%)	
Activity, n (%)			0.001*
A1	50 (72%)	16 (43%)	
A2	12 (17%)	19 (52.5%)	
A3	7 (10.5%)	2 (4.5%)	

Data are expressed by number (percentage), mean ± SD, or median (25%-75% percentiles) for viral load.

^a^ Data were analyzed by Mann Whitney *U*-test.

* indicates statistical significance (*P*<0.05).

### Serum miRNA expression levels in CHC patients and healthy controls

As there were no published results about expression levels of our selected miRNAs in serum of control and CHC patients, the expression of these miRNAs in control patients was first examined. All studied miRNAs were expressed in control serum with varying levels (Table 3). In CHC patients, serum expression levels of miR-34a, miR-130a, miR-19a, miR-192, miR-296 (each, *P*<0.0001), and miR-195 (*P* = 0.0002) were significantly higher than in healthy controls, corresponding to an average fold change (CHC/control) of 5.9, 3.4, 4.62, 2.96, 2.2, and 5.5, respectively. In contrast, serum miR-146a level was significantly lower (*P* = 0.011) in CHC by 2 fold comparing to controls (Table 3).

**Table 3:** Serum miRNA expression levels in CHC patients and healthy controls.

miRNA	CHC patients (n = 106)	Controls (n = 40)	*P* value
miR-146a	2.75 (1.07–13.62)	5.77 (5.14–22.75)	0.011*
miR-34a	3.54 (1.71–7.3)	0.61 (0.28–0.78)	<0.0001*
miR-130a	3.41 (1.73–10.7)	1.01 (0.12–1.03)	<0.0001*
miR-19a	6.28 (2.29–20.12)	1.36 (0.91–2.03)	<0.0001*
miR-192	1.91 (0.82–3.6)	0.64 (0.05–0.86)	<0.0001*
miR-195	21.56 (8.75–53.1)	9.8 (4.8–19.3)	0.0002*
miR-296	2.2 (0.89–3.82)	0.4 (0.2–0.62)	<0.0001*

Data are expressed as median (25%-75% percentiles) and were analyzed by Mann-Whitney *U* test.

* indicates statistical significance (*P*<0.05).

### Correlations between miRNAs expression and clinical data in CHC patients

Expression of miR-34a was significantly correlated with viral load (r = -0.22, *P* = 0.026) and AST levels (r = -0.21, *P* = 0.034) in reverse direction. miR-195 expression was negatively correlated with ALT (r = -0.23, *P* = 0.018) and AFP levels (r = -0.223, *P* = 0.022). There was a significant positive correlation between miR-192 expression and ALP activity (r = 0.2, *P* = 0.04). Expression of miR-19a was significantly correlated with platelet count (r = -0.232, *P* = 0.017) in reverse direction. There were no correlations between miR-146a, miR-130a, and miR-296 expression levels and clinical data.

### Correlations between studied miRNAs in CHC and healthy controls

Correlations between expression levels of our selected INF-related miRNAs were examined in control and CHC group. No correlations between miRNAs expression were found in the control group. However, in CHC group, several significant positive correlations between the tested miRNAs expression levels were found (Table 4). miR-19a expression was correlated with miR-146a (r = 0.35, *P* = 00.00), miR-130a (r = 0.282, *P* = 0.004), miR-192 (r = 0.352, *P* = 00.00), miR-195 (r = 0.5, *P*<0.0001), and miR-296 (r = 0.31, *P* = 0.0002) expression levels, respectively. Expression levels of miR-192 were correlated with those of miR-146a (r = 0.45, *P*<0.0001), and miR-296 (r = 0.54, *P*<0.0001), respectively. Expression of miR-130a was correlated with miR-146a (r = 0.26, *P* = 0.007), and miR-34a (r = 0.35, *P* = 00.00) expression levels, respectively.

**Table 4:** Significant correlations between studied miRNAs in CHC group.

miRNA	miR-146a	miR-34a	miR-130a	miR-19a	miR-192	miR-195	miR-296
miR-146a	-	-	r = 0.26	r = 0.35	r = 0.45	-	-
			*P* = 0.007	*P* = 00.00	*P*<0.0001		
miR-34a	-	-	r = 0.35	-	-	-	-
			*P* = 00.00				
miR-130a	r = 0.26	r = 0.35	-	r = 0.28	-	-	-
	*P* = 0.007	*P* = 00.00		*P* = 0.004			
miR-19a	r = 0.35	-	r = 0.28	-	r = 0.35	r = 0.5	r = 0.31
	*P* = 00.00		*P* = 0.004		*P* = 00.00	*P*<0.0001	*P* = 0.0002
miR-192	r = 0.45	-	-	r = 0.35	-	-	r = 0.54
	*P*<0.0001			*P* = 00.00			*P*<0.0001
miR-195	-	-	-	r = 0.52	-	-	-
				*P*<0.0001			
miR-296	-	-	-	r = 0.31	r = 0.54	-	-
				*P* = 0.0002	*P*<0.0001		

Associations between miRNA levels were determined in CHC patients (n = 106) using Spearman correlation. r: Spearman rho coefficient.

### Serum miRNA expression levels in SVR and NR groups to PEG-INF-α2b/RBV therapy

**Fig 1:**
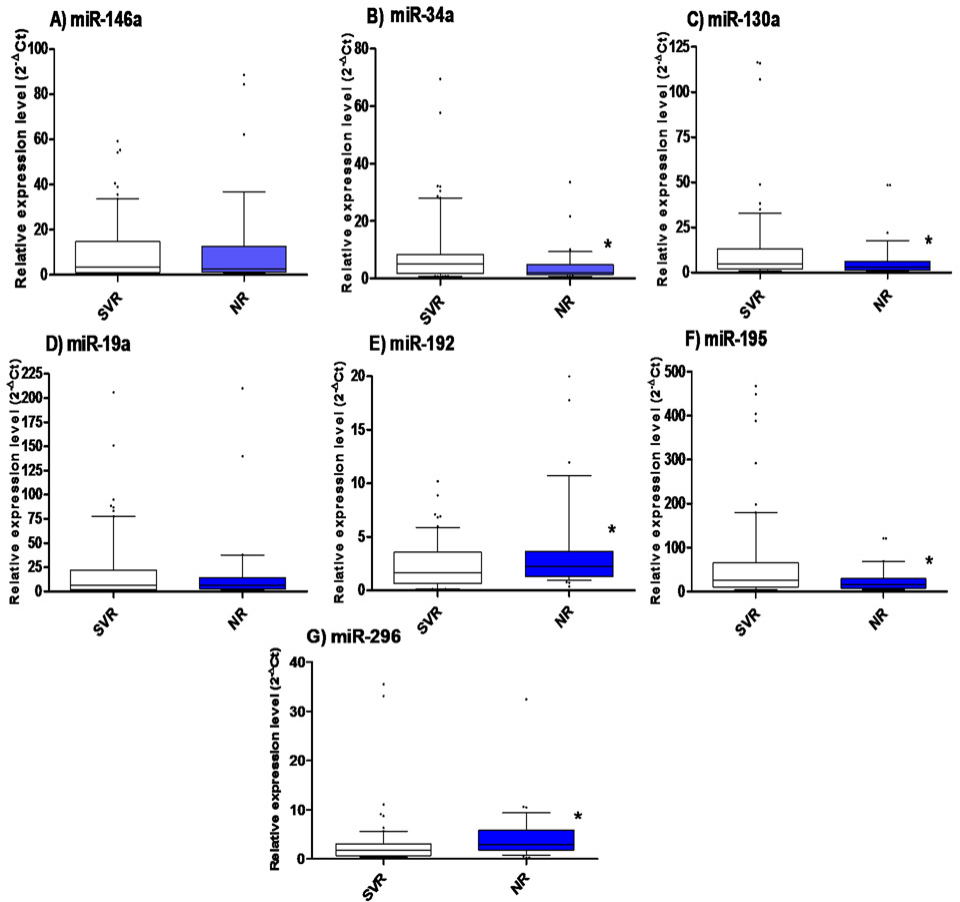
Serum miRNA expression levels in SVR and NR patients. The box represents the 25%-75% percentiles; the line inside the box represents the median and the upper and lower lines representing the 10%-90% percentiles of pretreatment expression levels of (a) miR-146a, *P* = 0.96, (b) miR-34a, *P* = 0.009, (c) miR-130a, *P* = 0.039, (d) miR-19a, *P* = 0.52, (e) miR-192, *P* = 0.035, (f) miR-195, *P* = 0.019, and (g) miR-296, *P* = 0.001 in SVR (n = 69) and NR patients (n = 37). Data were analyzed by Mann-Whitney *U* test. * indicates statistical significance (*P*<0.05).

Pretreatment expression levels of serum miR-34a, miR-130a, miR-192, miR-195, and miR-296 were differentially expressed in SVR and NR patients, whereas miR-146a and miR-19a expression levels were not significantly different (Fig. 1A-G). Levels of miR-34a (*P* = 0.009), miR-130a (*P* = 0.039), and miR-195 (*P* = 0.019) were significantly higher in SVR than NR patients, whereas levels of miR-192 (*P* = 0.035) and miR-296 (*P* = 0.001) were significantly higher in NR compared with SVR group.

### ROC curve analysis

**Fig 2:**
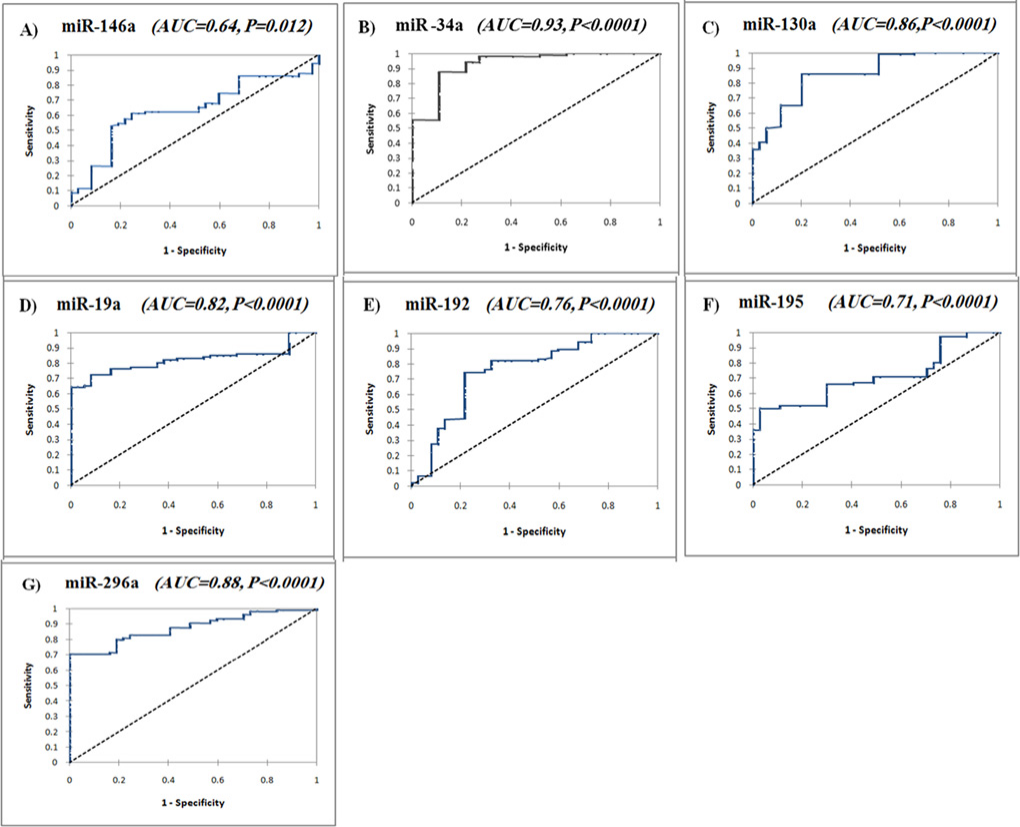
Serum INF-related miRNAs as diagnostic biomarkers in CHC. ROC curve analysis of serum miR-146a (a), miR-34a (b), miR-130a (c), miR-19a (d), miR-192 (e), miR-195 (f), and miR-296 (g) as diagnostic biomarkers differentiating CHC patients (n = 106) from healthy controls (n = 40).

ROC analysis (Fig. 2A-G) revealed that all of studied miRNAs could differentiate CHC patients from healthy controls with an AUC of 0.64 for miR-146a (95% CI 0.54–0.74, *P* = 0.012), 0.93 for miR-34a (95% CI 0.88–0.97, *P*<0.0001), 0.86 for miR-130a (95% CI 0.79–0.93, *P*<0.0001), 0.82 for miR-19a (95% CI 0.75–0.89, *P*<0.0001), 0.76 for miR-192 (95% CI 0.66–0.86, *P*<0.0001), 0.71 for miR-195 (95% CI 0.62–0.79, *P*<0.0001), and 0.88 for miR-296 (95% CI 0.82–0.93, *P*<0.0001), respectively. The optimal sensitivity and specificity to differentiate CHC from controls were (61.3% and 75.7% at a cutoff expression value <5.48) for miR-146a, (87.7 and 92.8% at a cutoff expression value >0.9) for miR-34a, (85.8 and 80.0% at a cutoff expression value >1.01) for miR-130a, (72.6 and 91.9% at a cutoff expression value >2.53) for miR-19a, (74.5 and 78.4% at a cutoff expression value >0.87) for miR-192, (50.0 and 97.3% at a cutoff expression value >21.6) for miR-195, and (70.5 and 100.0% at a cutoff expression value >1.2) for miR-296, respectively. These results suggest that all studied miRNAs could be potential diagnostic biomarkers in CHC.

Comparison of the ROC curve results suggested that the diagnostic accuracy of serum miR-34a was superior; in the order of miR-34a>miR-296>miR-130a>miR-19a>miR-192>miR-195>miR-146a (AUC = 0.93, 0.88, 0.86, 0.82, 0.76, 0.71, and 0.64, respectively). Although miR-19a was more upregulated than miR-130a (4.6 versus 3.4 folds, respectively) in CHC, miR-130a showed higher diagnostic accuracy than miR-19a (AUC 0.86 versus 0.82, respectively).

**Fig 3:**
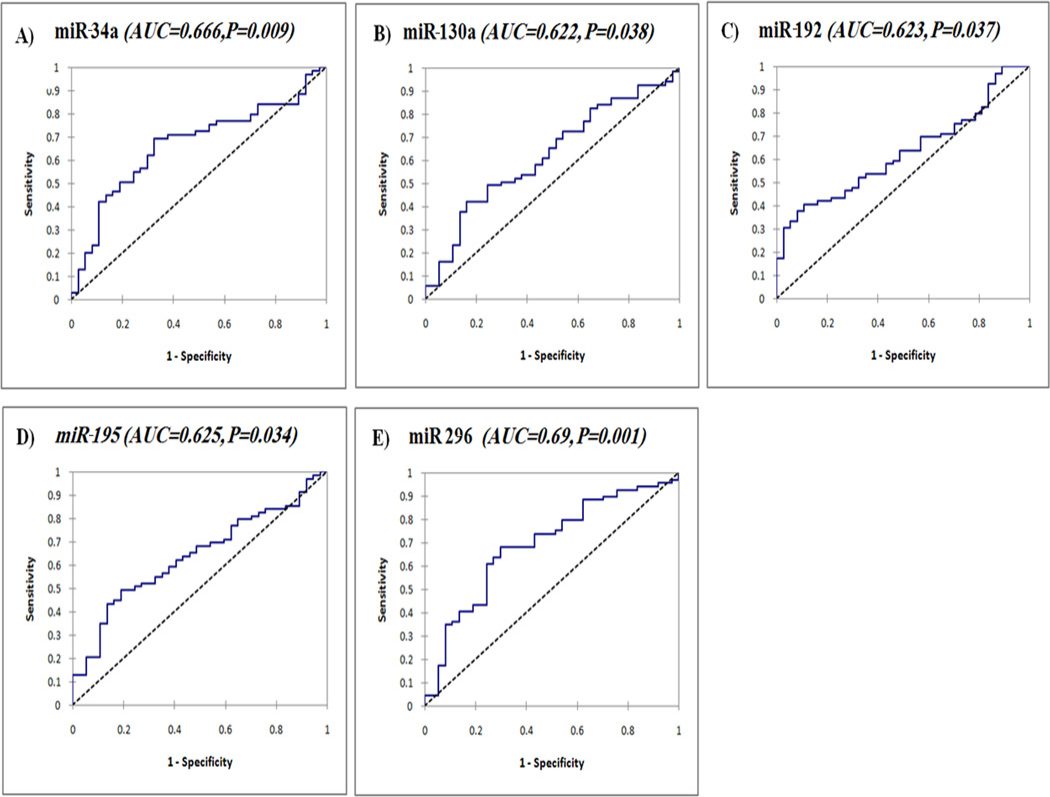
Serum INF-related miRNAs as prognostic biomarkers for treatment outcome in CHC. ROC curve analysis of serum miR-34a (a), miR-130a (b), miR-192 (c), miR-195 (d), and miR-296 (e) as prognostic biomarkers discriminating SVR (n = 69) from NR patients (n = 37).

The prognostic significance of serum miR-34a, miR-130a, miR-192, miR-195, and miR-296 which were differentially expressed in SVR and NR groups was assessed using a ROC curve (Fig. 3A-E). The results revealed that these miRNAs could discriminate between SVR and NR among CHC patients with AUC of 0.666 for miR-34a (95% CI 0.55–0.76, *P* = 0.009), 0.622 for miR-130a (95% CI 0.51–0.73, *P* = 0.038), 0.623 for miR-192 (95% CI 0.52–0.75, *P* = 0.037), 0.625 for miR-195 (95% CI 0.52–0.73, *P* = 0.034), and 0.69 for miR-296 (95% CI 0.58–0.79, *P* = 0.001), respectively. The optimal sensitivity and specificity to differentiate SVR from NR patients were (67.3 and 68.2% at a cutoff expression value >2.48) for miR-34a, (51.3 and 65.2% at a cutoff expression value >2.97) for miR-130a, (51.3 and 63.77% at a cutoff expression value <2.2) for miR-192, (59.5 and 60.9% at a cutoff expression value >17.2) for miR-195, and (70.3 and 68.2% at a cutoff expression value <2.37) for miR-296, respectively. The prognostic significance of clinical data showed AUC of 0.65 (95% CI 0.54–0.76, *P* = 0.007) for ALT, 0.67 (95% CI 0.57–0.79, *P* = 0.002) for viral load, and 0.66 (95% CI 0.55–0.78, *P* = 0.003) for AFP, with sensitivity and specificity of 65.2% and 64.9%, 40.5% and 94%, and 68% and 65%, respectively.

Comparison of the ROC curve results regarding studied miRNAs suggested that the prognostic accuracy of pretreatment serum miR-296 was superior; followed by miR-34a, with an AUC 0.69 and 0.666, respectively, whereas the prognostic accuracy of serum miR-130a, miR-192, and miR-195 was nearly the same with an AUC of 0.62.

### Logistic regression analysis

Univariate and multivariate logistic regression analyses were performed regarding the outcome variables in response to combination therapy. ALT, AST, ALP, AFP, platelet count, and expression levels of miR-34a, miR-192, and miR-195 were selected as significant predictor variables of response in the univariate analysis (Table 5). In the multivariate analysis, AFP, platelet count, and expression levels of miR-34a, miR-192, and miR-195 were selected as significant independent variables that could predict the response to PEG-IFN-α2b/RBV therapy (Table 5). Interestingly, miR-34a, miR-195 levels and platelet count were positive predictors for SVR, whereas AFP and miR-192 were independent negative variables affecting the response.

**Table 5:** Factors that predict response to PEG-INF-α2b/RBV therapy using logistic regression analysis.

Variables	B	S.E.	*P* value	Odds ratio	Odds ratio (95% CI)
***Univariate logistic regression analysis***					
ALT (IU/l)	-0.262	0.117	0.025*	0.981	(0.966–0.998)
AST (IU/l)	-0.332	0.134	0.013*	0.973	(0.953–0.994)
ALP (IU/l)	-0.319	0.121	0.008*	0.989	(0.980–0.997)
AFP (ng/ml)	-0.335	0.121	0.006*	0.846	(0.752–0.952)
Platelet count	0.354	0.141	0.012*	1.011	(1.002–1.020)
miR-34a	0.446	0.22	0.045*	1.073	(1.002–1.153)
miR-192	-0.256	0.125	0.04*	0.868	(0.758–0.994)
miR-195	0.617	0.307	0.044*	1.013	(1–1.026)
***Multivariate logistic regression analysis***					
AFP (ng/ml)	-0.393	0.159	0.013*	0.822	(0.704–0.968)
Platelet count	0.392	0.189	0.038*	1.013	(1.001–1.025)
miR-34a	0.847	0.309	0.006*	1.151	(1.041–1.272)
miR-192	-0.525	0.194	0.007*	0.750	(0.605–0.925)
miR-195	0.607	0.308	0.048*	1.013	(1–1.025)

S.E.: standard error.

* indicates statistical significance (*P*<0.05).

## Discussion

Understanding pretreatment miRNA profiles associated with therapeutic response to standard therapy for CHC would be a critical pharmacogenetic tool for personalizing therapy for NR patients. To the best of our knowledge, no one has examined the expression profiles of serum INF-related miRNAs in CHC genotype 4 patients and their relation to virological response to INF-based therapy. HCV-4 was chosen for this investigation since it is the predominate genotype in Egypt, resistant to treatment, and responsible for the majority of HCV infections worldwide, as previously reported [9,32].

We used real-time PCR custom array technology as a powerful tool to estimate serum miRNAs. Quantitative PCR was advantageous over microarray by its high sensitivity, accuracy, lower cost, and lower sample requirements [33]. All studied miRNAs were differentially expressed in sera from CHC patients and could discriminate CHC from controls by ROC analysis, suggesting that these miRNAs could serve as surrogate markers of CHC. Our results coincide with previous reports showing changes in circulating miRNAs in serum during HCV infection [13,34]. Differentially regulated serum miRNAs in HCV would most probably originate from the liver due to hepatic pathology [34]. miRNAs are associated with exosomes as well as RNA-binding protein complexes [11]. Liver and serum miRNA levels were correlated, but some miRNAs showed pathogen-specific serum profiles [35]. Serum miR-122 was shown to be a surrogate for hepatic miR-122 [21]. Thus, changes in hepatic miRNA profiles upon HCV infection could be reflected in the serum. This may highlight the use of circulating miRNAs as non-invasive biomarkers in CHC.

Our data revealed significant correlations between studied miRNAs in CHC patients but not in healthy controls. These results suggest concomitant or concordant expression of INF-related miRNAs in response to HCV infection. Whether this concomitant expression of these miRNAs being HCV-specific yet to be investigated. It is likely that at least some of the investigated miRNAs are associated with HCV pathogenesis or play a role in host defense against viral infection.

The present study revealed that 65% of the patients treated with PEG-IFN-α2b/RBV have achieved SVR, as comparable to earliarly reported [36,37]. Pretreatment expression of five INF-related miRNAs; miR-34a, miR-130a, miR-195, miR-192, and miR-296 were associated with the outcome of combination therapy. Interestingly, multivariate analysis revealed higher pretreatment miR-34a and miR-195 as predictors for SVR, whereas increased pretreatment miR-192 could be a predictor of non-response among CHC patients, implying that these miRNAs could be used as genetic predictors of treatment outcome.

Our results demonstrated that miR-34a showed the highest upregulation with the highest discriminating power (AUC = 0.93) to differentiate CHC patients from controls, implicating miR-34a as a powerful diagnostic biomarker of CHC. A pronounced upregulation of serum miR-34a was previously observed in CHC patients and was correlated with inflammation activity, fibrosis stage, and disease progression [13]. However, these correlations were not found in our study. miR-34a upregulation may be attributed to its induction by IFN-γ released by immune cells in response to HCV infection. This IFN-induced miRNA is transcriptionally activated, or expressed indirectly as a result of the expression of IFN-inducible proteins [26]. miR-34a is a central mediator of p53 function, and may indirectly inhibits viral replication via induction of apoptosis among virus-infected cells [38]. miR-34a was upregulated by interleukin (IL)-1β mediating the IL-1β-induced expression of inducible nitric oxide synthase, a key ISG, limiting virus replication in macrophages by inducing macrophage apoptosis via nitric oxide [39]. This favorable role may in part explain association of miR-34a with SVR. In addition, miR-34a may target and downregulate hepatocytes nuclear factor-4α, which was shown to induce multidrug resistance proteins [16].

The observed significant negative correlation between miR-34a and viral load may also explain its association with SVR; where SVR were demonstrated to have lower HCV RNA than NR group. This correlation, despite weak, may also in part indicate a mutual relationship between HCV and miR-34a. miR-34a expression was increased [13], or decreased [16] in different HCV models. However, whether it is being directly altered or altering the virus yet to be studied. We observed that miR-34a was negatively correlated with AST levels. In contrast, a previous study reported a positive correlation [13].

The present study demonstrated significant upregulation of miR-296 in CHC. miR-296 was increased in PBMCs [23], or decreased in liver biopsy from CHC patients [40]. Controversial results between miRNAs in serum and liver tissue have been observed in drug-induced liver injury [12]. However, there are several reports of miRNAs that are upregulated in the serum as well as infected or cancerous tissue [21,41]. Meanwhile, miR-296 was induced by IFN-β treatment and inhibited HCV replication in human hepatoma cell line (Huh7) by directly targeting the viral RNA [15]. miR-296 was also induced by INF-α treatment in human PBMCs of healthy individuals as well as CHC patients [23]. Thereby, miR-296 may be a potential strategy to protect against liver injury during CHC. It is unknown whether HCV has evolved a counter mechanism to this antiviral miRNA, although some studies suggest that such a mechanism might exist [40].

Pretreatment expression of miR-296 was observed to be associated with non-response, with the highest prognostic power (AUC = 0.69) and was superior to ALT, viral load, and AFP (AUC = 0.65, 0.66, and 0.67, respectively), suggesting its potential for future use to discriminate SVR and NR patients. The relation between elevated pretreatment levels of miR-296 and non-response may be explained on the basis that pretreatment upregulation of ISGs in the liver was associated with non-response to INF therapy, in part due to induction of refractory state to further stimulation by exogenous INF [42].

In agreement with our results, miR-192, a liver specific miRNA [25], showed increased expression in sera in different etiologies associated with liver injury [12,43–45]. miR-192 was induced by INF-α in Huh cells [17,25] and downregulated upon HCV infection [17]. However, its role in INF defense mechanism is unknown. Interestingly, miR-192/miR-215 expression was upregulated by HCV infection and enhanced the replication of HCV replicon cells as well as HCV itself [46]. This may explain the observed relation between pretreatment serum miR-192 and failure of response. In contrast, hepatic miR-192 expression was slightly higher in SVR when compared with non-SVR [20]. These conflicted results may be due to difference in HCV genotypes.

Despite that the observed prognostic accuracy of miR-192 (AUC = 0.62) was lower than viral load, ALT and AFP, which were also associated with non-response, multivariate analysis revealed only serum miR-192 and AFP as independent variables for non-response. Previous results have shown pretreatment viral load and AFP to associate with non-response with AFP being an independent predictor of treatment failure [37]. The observed positive correlation between miR-192 and ALP may suggest release of miR-192 during bile duct inflammation associated with CHC.

Significant upregulation of serum miR-130a in CHC was observed. Similarly, miR-130a was upregulated in HCV Con-1 replicon [16], HCV-infected hepatocytes [47], and in liver biopsy from CHC patients [40,47], thus, these could be reflected in the serum. In contrast, miR-130a was downregulated upon acute HCV infection to Huh7.5 cells [17,48], or unchanged in HCV-expressing Huh7 cells [49]. The discrepancies in miRNA expression profiles between different studies may be due to different *in vitro* models and cell types, or different HCV genotypes.

miR-130a was induced by INF-α treatment in Huh cells and in HCV-infected hepatocytes [17,25] and was shown to potentially regulate HCV [40]. miR-130a/301 may interfere with the INF pathway by targeting interferon regulatory factor-1 and STAT3 genes in HCV-infected cells [16]. miR-130a was predicted to target endocytosis and transforming growth factor-β signaling, that affect cell growth and apoptosis [17]. miR-130a was upregulated by HCV and facilitated HCV replication by targeting antiviral interferon inducible trans-membrane protein (IFITM); knockdown of miR-130a upregulated IFITM expression and inhibited HCV replication in hepatocytes [47]. Similarly, miR-130a augmented HCV replication in infected cells as anti-miR-130a downregulated HCV replication [17]. Conversely, miR-130a downregulated HCV replication by restoring the innate immune response [50]. The exact mechanism by which HCV modulates miR-130a expression or how miR-130a alters HCV replication is presently not well known.

Our study demonstrated that miR-130a expression was associated with SVR. This could be due to restoration of innate immune response by miR-130a upregulation which helps viral clearance [50]. miR-130a upregulation mediated INF-α-induced downregulation of the proto-oncogene, c-Met, and inhibition of viral pathogenesis [17].

Serum miR-195 and miR-19a were differentially expressed in HBV-infected samples comparing to controls in microarray [44], however, to the best of our knowledge there are no reports about miR-195 and miR-19a expression in CHC. Taking this advantage, we examined their serum expression levels and found upregulation of miR-195 and miR-19a in CHC. miR-195 was induced by INF-β in activated hepatic stellate cells; INF-β showed antiproliferative and antifibrotic effects independent of its viral effect by upregulating miR-195 which targets cyclin E1 expression leading to cell cycle arrest [24]. miR-19a was induced by INF-α treatment to Huh7 cells [25,51], and activated the INF pathway and IL-6 pathway by inhibiting suppressor of cytokine signaling SOCS3 expression thus activating JAK/STAT signaling, implicating miR-19a in the regulation of both antiviral and pro-inflammatory responses [51]. This suggests that the IFN-α-induced miR-19a may modulate gene expression in hepatocytes during HCV infection.

The present study revealed that pretreatment miR-195 expression was associated with SVR, whereas the response to combination therapy was not affected by baseline miR-19a. Multivariate analysis revealed pretreatment miR-195 as an independent predictor for SVR. This relation may be due to miR-195-mediated INF-β antifibrotic effect [24]. However, we found no correlation between miR-195 expression and fibrosis score, but this may be partially supported by the observed negative correlations of miR-195 expression with ALT, a hepatic necroinflammatory marker, and AFP, a marker of disease progression and interferon resistance. Interestingly, miR-19a was negatively correlated with platelet count. Platelet count was associated with SVR in our study and favorably predicts SVR in multivariate analysis. However, this association remains to be investigated.

The current study demonstrated reduced levels of serum miR-146a in CHC. Similarly, miR-146a was downregulated in HCV Con1 replicon [16]. In contrast, miR-146a was not changed in PBMCs from HCV-4 patients [52], or upregulated in serum of CHC patients [53]. Serum pretreatment miR-146a expression wasn’t correlated with treatment response in our study. Similar results were obtained from PBMCs of CHC patients [52]. miR-146a plays pivotal roles in the innate immune response by regulating the NF-κB pathway [54]. miR-146a is induced downstream of the Toll-like receptor and the INF-α/β pathways and regulates the INF pathway by negative feed-back inhibition of TNF receptor-associated factor 6 (TRAF6), IL-1 receptor associated kinase (IRAK2), and STAT1 proteins [54]. Repressed induction of miR-146a suggests that the INF-related miR-146a is altered by HCV, which may be due to HCV-induced interference with the Toll-like receptor signaling [52].

Finally, our study is limited by the relatively low number of patients; however, it suggests that five INF-related miRNAs were associated with virological response to INF/RBV therapy and may stimulate other studies to fully characterize their cellular targets and their modes of action.

## Conclusions

All studied miRNAs were differentially expressed in serum of CHC patients and could differentiate CHC patients from controls, implicating these miRNAs as potential biomarkers for CHC, with miR-34a being of superior diagnostic accuracy. Correlations between expression levels of studied miRNAs suggested concomitant expression of INF-related miRNAs in response to viral infection, implicating these miRNAs as positive or negative regulators of HCV and as key players in HCV pathogenesis. Five of studied miRNAs were associated with virological response to standard INF therapy and could discriminate SVR from NR patients, with miR-296 being of superior prognostic accuracy. Increased pretreatment levels of miR-34a and miR-195 were independent predictors of SVR, while miR-192 upregulation could be an independent variable for non-response. Our miRNA profiling results could be implicated as novel non-invasive diagnostic and prognostic pharmacogenetic biomarkers for treatment personalization in CHC and could be used to identify new miRNA-based antivirals.
